# Immunometabolic reprogramming: a working framework for disease development, endotype differentiation, and therapeutic considerations in pediatric asthma

**DOI:** 10.3389/fimmu.2026.1999531

**Published:** 2026-10-19

**Authors:** Kaiwen Zheng, Ran Ma, Yingqian Zhang

**Affiliations:** 1 Graduate School, Hebei Medical University, Shijiazhuang, Hebei, China; 2 Department No. 3 of Respiratory Medicine, Hebei Children’s Hospital, Shijiazhuang, Hebei, China; 3 Hebei Provincial Clinical Research Center for Child Health and Disease, Hebei Children’s Hospital, Shijiazhuang, Hebei, China; 4 Hebei Key Laboratory of Pediatric Allergy and Immunology, Hebei Children’s Hospital, Shijiazhuang, Hebei, China

**Keywords:** endotype, ferroptosis, glucocorticoid resistance, immunometabolism, metabolic reprogramming, obese asthma, pediatric asthma, trained immunity

## Abstract

**Background:**

Paediatric asthma exhibits marked clinical heterogeneity and cannot be simply considered the childhood counterpart of adult asthma. Early-life environmental exposures can reshape immune-cell metabolic programs and potentially drive long-lasting inflammatory phenotypes. Existing explanatory frameworks for paediatric asthma largely centre on Th2-related inflammation, epithelial-alarm signalling and neuro-immune crosstalk, while the contributions of immunometabolic processes to disease endotypes remain under-summarised.

**Objective:**

This review collates available evidence regarding metabolic reprogramming in the pathogenesis of paediatric asthma and provides an integrated immunometabolic perspective on this condition.

**Results:**

Summarized evidence covers immunometabolic changes across key airway-resident and infiltrating cell populations. Discussion addresses core paediatric-specific biological features, including early-life developmental windows, virus-related trained immunity, obese asthma, circadian-sleep interactions, neuro-metabolic-immune communication and metabolomics-based endotyping. The framework is organized around substrate competition rather than a glycolysis-versus-OXPHOS binary, because Tregs, M2-like macrophages and pathogenic ILC2s share lipid and amino-acid pools but exert opposing functions. From these observations emerges a testable “Metabolic Latching Model” of how early-life insults establish persistent inflammatory memory through metabolism–epigenetic coupling, with “latching” operationally defined by exposure removal and persistence beyond responding-cell turnover. Group 2 innate lymphoid cells (ILC2s) and the Treg-to-effector balance are placed at the centre of this framework. Three hypotheses are prioritised as feasible with current technology: H1 (monocyte glycolytic baseline predicting wheeze-to-asthma transition), H4 (metabolic intervention in obese asthma), and H6 (the HDAC2-GSH/butyrate axis in steroid sensitivity).

**Conclusion:**

Current evidence suggests that immunometabolic reprogramming serves as one plausible contributing factor for the heterogeneity of paediatric asthma. This conceptual framework may bridge molecular metabolic findings and paediatric clinical contexts, and inform future mechanistic and clinical studies in this research area.

## 1 Introduction: rationale for a metabolic perspective in paediatric asthma

The global prevalence of paediatric asthma keeps rising, and patient manifestations vary greatly. Some children only have temporary wheeze triggered by viruses. Other children develop persistent severe disease that responds poorly to steroids (1). Traditional classification systems rely on Th2 status or eosinophil-neutrophil inflammation. These systems cannot fully explain disease diversity. They also cannot explain how early-life exposures lead to distinct inflammatory endotypes years later.

Paediatric asthma is not a single disease but a set of trajectories. Longitudinal birth cohorts show that early-life wheeze partitions into distinct pathways: transient wheeze that resolves by school age, persistent wheeze that continues into adulthood, and late-onset wheeze (2, 3). Which pathway a child follows is the central clinical question, and it is precisely the question a metabolic framework must explain: the Metabolic Latching Model proposes, in part, that trajectory divergence reflects whether early-life metabolic-epigenetic latching is established, and whether it is later resolved (Section 6).

Metabolic reprogramming means adaptive changes in cellular substrate use and energy metabolism after external stimulation. It is well documented in cancer research (4). Studies in immunometabolism show that immune cell function depends heavily on metabolic states. Metabolic intermediates can act as substrates for epigenetic modifiers. They form regulatory loops linking cell signals, metabolism and epigenetic modification. These loops may help explain how early-life environmental exposures contribute to asthma endotypes in children.

Children have three important biological characteristics. First, a critical developmental window exists roughly from birth to six years old (2). Lung structure, immunity and metabolic homeostasis mature rapidly during this period. Metabolic disturbances in this window may cause long-lasting biological outcomes. Second, immune cell metabolism in children shows higher plasticity than in adults, an inference from the trained-immunity literature (Section 2.6) rather than a directly measured quantity, which itself marks how much basic paediatric immunometabolism remains unmeasured. This feature may create opportunities for early intervention. Third, childhood metabolic programming comes from both external environment and prenatal conditions. Prenatal nutrition, delivery mode and early feeding habits all play a role (Section 4.1).

Direct human paediatric data on immunometabolism remain limited. For this reason, this review follows a clear structure. First, we introduce basic immunometabolic mechanisms shared across different age groups (Sections 2-3). Next, we describe how these pathways function under paediatric biological backgrounds (Section 4). Finally, we discuss the value and practical limits of metabolic-targeted therapies for children (Section 5). Many metabolic pathways work similarly across the lifespan. However, their modification in developmental windows can shape long-term asthma trajectories. This supports the value of studying paediatric asthma to understand asthma across age groups.

The so-called “fourth dimension” of metabolic regulation works together with the classical Th2 inflammatory, epithelial-alarm and neuro-immune axes. It does not replace these existing frameworks. The neuro-metabolic-immune crosstalk described in Section 4.7 reflects metabolic adjustment of known neuro-immune signals. It is not an independent regulatory axis. The three classical axes mainly answer which cells produce inflammation. Metabolic signatures may help us understand why inflammation persists. Its explanatory value is greatest when it explains which cell wins under limiting substrate conditions—particularly ILC2s, Tregs and M2-like macrophages, which share lipid-oxidative and amino-acid-dependent programmes but exert opposing functions in paediatric asthma. Therefore, this review summarizes existing literature and puts forward a testable working model requiring further experimental verification (Section 6).

## 2 General principles of immune cell metabolic reprogramming

### 2.1 Glycolysis versus oxidative phosphorylation: beyond simple binary classification

Pro-inflammatory immune cells such as M1-like macrophages, Th1/Th17 cells and activated mast cells often use aerobic glycolysis, also known as the Warburg effect. This process is controlled by HIF-1α, mTOR and PI3K-Akt signals. In contrast, reparative or tolerogenic cells including M2-like macrophages, Treg cells and memory T cells tend to use fatty acid oxidation (FAO) and oxidative phosphorylation (OXPHOS). This general pattern has been repeatedly observed in asthma-related research (5). Still, several important points need consideration (6).

Cell metabolic states form a continuous spectrum, not simple on-off switches. Many intermediate phenotypes exist between typical M1 and M2 states (Section 3.1). Glycolysis mainly supports biosynthesis and intracellular signal transmission. It is not only used for ATP production. For example, NADPH is generated mainly by the pentose phosphate pathway branching from glucose-6-phosphate, not by glycolysis per se. Succinate accumulation that stabilizes HIF-1α is a TCA-cycle event (7), demonstrated primarily in mouse and human macrophage models. Lactate acts both as a metabolic product and a signal molecule. Histone lactylation controlled by lactate can directly change gene transcription. This mechanism may support chronic airway inflammation (8). This lactate-dependent programme is also relevant to Treg biology: Foxp3 shifts Tregs away from Myc-driven glycolysis toward oxidative metabolism, allowing them to function in lactate-rich conditions that suppress effector T cells (Section 3.3).

A caveat qualifies the glycolysis-versus-oxidative dichotomy throughout this review. Three populations with opposite roles in this disease, Tregs, M2-like macrophages and pathogenic ILC2s, converge on the same oxidative-lipid programme: Tregs run on lipid oxidation, alternatively activated macrophages depend on lipid uptake and lysosomal lipolysis, and ILC2s draw on the same fatty-acid pool for FAO and lipid-droplet formation (Section 3.2). The useful question is therefore which cell captures the substrate when fuel is limiting in an inflamed airway, a competition that pathway-preference data cannot answer and that carries direct consequences for the therapeutic logic of Section 5.

### 2.2 Lipid mediators: substrate competition in the arachidonic acid cascade

Cells produce arachidonic acid after breaking down membrane phospholipids. This substance flows into LOX or COX pathways. These two sets of pathways balance the production of pro-inflammatory lipid mediators (leukotriene B4, cysteinyl leukotrienes) and pro-resolving lipid mediators (resolvins, protectins). Studies in asthma models report impaired cholesterol efflux controlled by ABCA1/G1 and foam-cell-like macrophage changes (6).

Lipid mediators may serve as measurable markers of metabolic reprogramming. Lipidomic tests on exhaled breath condensate or induced sputum may reflect disease endotypes more stably than cytokine detection. This idea still needs full verification in paediatric patient cohorts.

### 2.3 Ferroptosis: potential effects of lipid metabolic changes

Ferroptosis is iron-dependent regulated cell death driven by phospholipid peroxidation. Its core protective system includes the GPX4-glutathione (GSH) pathway. System Xc- cystine-glutamate exchanger and FSP1-CoQ10 pathway also provide protection (9, 10). Ferroptosis-related mechanisms may affect asthma in several ways.

In airway epithelium, low GPX4 activity and excess lipid peroxidation may damage epithelial barriers and increase goblet cell metaplasia. Air pollutants such as PM2.5 and ozone can trigger epithelial ferroptosis in cell systems and animal models (11). In neutrophil-dominant inflammation, neutrophils face high redox pressure. NETosis shares partial mechanisms with ferroptosis, including lipid peroxidation and NADPH consumption; this mechanism is derived mainly from mouse and *in vitro* neutrophil studies, with human data limited to associations. The two processes may work together as terminal effector pathways.

Studies of type-2 inflammation reveal a lymphocyte-related paradox. Type-2 innate and adaptive lymphocytes, rather than eosinophils, acquire ferroptosis tolerance as an active adaptation. Wientjens and colleagues showed that cystine uptake sustains glutathione synthesis in ILC2s and Th2 cells; GPX4 and TXNRD1 rise, ferroptosis tolerance follows, and lipid droplet accumulation is downstream of that tolerance. The tolerance itself facilitates pathogenic type-2 immunity in allergic airway inflammation (12). On the other side, experimental induction of eosinophil ferroptosis-like death enhances anti-inflammatory effects of glucocorticoids (13, 14). Whether strategies targeting ferroptosis can be used for paediatric asthma requires further study (12–14).

### 2.4 Amino-acid and one-carbon metabolism

Three amino-acid related pathways show abnormal activity in asthma models. In arginine metabolism, iNOS produces NO while ARG1 produces ornithine. The two reactions compete for substrates. In tryptophan metabolism, the IDO-kynurenine pathway affects Treg function and nerve signal transmission. In glutaminolysis, metabolic flow supports TCA cycle supplementation and mTOR activity (15, 16).

The tryptophan-kynurenine axis deserves attention in paediatric research. Gut microbiota strongly change tryptophan turnover. It forms a bridge connecting microbiome, metabolism and immunity (Section 4.4).

### 2.5 Mitochondrial quality control and inflammasomes: linking metabolic disturbance to inflammation

Metabolic reprogramming cannot be separated from organelle homeostasis. Mitochondrial quality control includes mitophagy, fission-fusion balance and PGC-1α-driven biogenesis. These processes set cell membrane potential and basic ROS levels.

When homeostatic systems fail, damaged mitochondria build up and release ROS. The NLRP3 inflammasome assembles and secretes IL-1β and IL-18. Leaked mitochondrial DNA activates the cGAS-STING pathway. It induces type-I interferon response and SASP-like cell status (17, 18).

Respiratory viruses can interfere with mitophagy. For example, RSV NS1 protein can hijack host mitophagy to escape type-I interferon defence. RSV infection also suppresses PINK1/Parkin-dependent mitophagy and accumulates damaged mitochondria (19, 20). Viruses can reshape host immunometabolism by changing mitochondrial quality control. This represents one virological feature of trained-immunity-like responses.

These mechanisms connect metabolic disturbance to classic inflammatory signals. They form part of the molecular basis for the Metabolic Latching Model introduced in Section 6. Compounds that induce mitophagy include urolithin A and spermidine. Their effects in paediatric airway inflammation remain largely unexplored.

### 2.6 Metabolic-epigenetic coupling: possible mechanisms for inflammatory memory

Metabolic intermediates such as acetyl-CoA, SAM, α-ketoglutarate, succinate and NAD+ work as substrates or regulators. They control histone acetylation, DNA methylation, demethylation, lactylation and deacetylation. Changes in cell metabolism can remodel chromatin without changing DNA sequences. This forms one molecular basis for trained immunity (21, 22).

Transient stimuli such as RSV infection, low-dose LPS or BCG vaccination can trigger metabolic changes through mTOR/HIF-1α. Innate immune cells maintain enhanced response ability long after stimuli disappear (23, 24). Existing reviews have summarized the interaction between metabolism and epigenetics in trained immunity (24). This axis may partly explain clinical phenomena such as priming effects after virus-caused wheeze in children. Still, direct human in-vivo evidence remains limited.

## 3 A cell-by-cell evidence map with notes from single-cell studies

Throughout Sections 2–3, we annotate the dominant evidence base of each claim: human clinical or cohort data (H), animal models (A), and cell-based or mechanistic studies (M). Most mechanistic metabolic findings remain at the A/M level; human airway data, where available, are noted explicitly.

### 3.1 Alveolar macrophages: possible central hub for airway metabolic reprogramming

Alveolar macrophages (AMs) are the most common immune cells inside airway lumen. They sit at the mucosal interface and contact inhaled irritants such as ozone, NO_2_, particulate matter and allergen protein. AMs with glycolysis bias show M1-like pro-inflammatory features and secrete high levels of IL-1β, IL-6 and TNF. AMs relying on FAO show better phagocytic clearance and immune homeostasis (6).

Public BALF single-cell datasets can help us test two questions. First, whether AM and T-cell transcription profiles overlap widely. Second, whether MHC-II-high activated AM subsets appear under inflammatory microenvironments. If we observe these patterns, it supports the idea of metabolism-epigenetics-driven cell functional continua instead of technical sequencing noise. This prediction carries weight only after doublet detection, ambient-RNA correction and protein-level confirmation (CITE-seq or flow cytometry): transcriptional overlap in inflamed BALF is otherwise indistinguishable from doublets and ambient mRNA, and the AM–T continuum claim must survive these filters before it can be read as biology.

We put forward a testable hypothesis. Alveolar macrophages may exist in at least three metabolic states. They include OXPHOS-dependent surveillance state, glycolysis-driven inflammatory state, and FAO-biased reparative state. Persistent paediatric asthma may relate to failure to produce reparative AMs or stable lock of glycolytic inflammatory AMs. Public single-cell transcriptome data combined with metabolic pathway activity scoring tools such as scMetabolism can test this hypothesis.

Counter-evidence deserves equal weight. Alveolar glucose concentration is below 10% of blood levels, and tissue-resident alveolar macrophages show almost no glycolytic reserve: they do not increase glycolysis after LPS and do not alter cytokine output when glycolysis is blocked, in contrast to bone-marrow-derived macrophages from which most of the M1-glycolysis literature derives (25). The resident-versus-recruited distinction matters most for the paediatric argument made here, because early-life exposures act predominantly on fetally derived resident macrophages whose metabolic wiring may differ fundamentally from recruited monocyte-derived cells. We therefore soften the corresponding claim in **Table 1**: alveolar macrophages are one candidate latching hub, not an established one.

**Table 1:** Overview of metabolic reprogramming across major cell types.

Cell type	Key metabolic alteration	Functional consequence	Paediatric relevance	Candidate intervention targets
Alveolar macrophage [A/M; H scRNA emerging]	Glycolytic bias/failure of the FAO reparative state	Pro-inflammatory amplification, impaired clearance	One candidate latching hub under repeated viral exposure (resident-versus-recruited distinction unresolved)	mTOR, HIF-1α, mitophagy induction
Effector T cells (Th2/Th17) [A/M; H ex vivo]	FASN and glutaminolysis dependence; Glut1-high glycolysis after activation	Type 2 inflammation maintenance	Sensitization and persistent inflammation in school-age children	mTOR inhibition, glutaminase inhibition
Treg [A/M; H ex vivo]	Lipid oxidation; Foxp3-driven oxidative programme, lactate-tolerant	Immune homeostasis; Treg-to-effector imbalance and Treg instability promote chronicity	Oral tolerance induction window in early life	Preserve Treg stability without global oxidative-metabolism push (compartment-specific; effects on ILC2/M2 substrate competition must be evaluated)
ILC2 [A; H ex vivo]	Arg1 checkpoint-dependent glycolysis; context-dependent FA metabolism and IL-33-imprinted lipid-droplet formation; cystine-GSH-GPX4 ferroptosis tolerance	Dominant innate IL-5/IL-13 source; steroid resistance via TSLP-STAT5	Central to early-life IL-33 window, viral wheeze, obese asthma	glycolysis/glutaminolysis blockade (with substrate-competition caveat, Section 2.1)
Mast cell [M; A]	FcϵRI-triggered burst glycolysis	Degranulation, EIB	Allergen challenge and exercise-induced wheeze	Glycolysis inhibition, mediator synthesis blockade
Airway epithelium [M/A; H ex vivo]	Glycolysis-alarm coupling, compromised GPX4 defence	Barrier breakdown, IL-33/TSLP release	First-line interface for pollutants and viruses	GPX4 induction, butyrate (HDAC2)
Airway smooth muscle [H tissue; A/M]	Stage-specific FAO and OXPHOS dependence, mitochondrial dysfunction	AHR, hypertrophy	Direct cellular substrate for diagnostic airway-hyperresponsiveness phenotypes	AMPK activation, metabolism-epigenome reset
Fibroblast [A/M]	Glycolysis-collagen coupling, glutamine-to-proline flux	Subepithelial fibrosis	Contributes to chronicity and long-term lung-function trajectories	Proline/hydroxylase axis
Eosinophil [M/A; H ex vivo]	OXPHOS-lipid axis dependence	Terminal type-2 inflammation	Key effector cells for anti-IL-5-directed biologics	IL-5 axis (already clinically deployed)
Neutrophil [H; A/M]	Extreme glycolysis, NADPH depletion, ferroptosis susceptibility	Severe disease, steroid resistance	Severe or infection-provoked exacerbations	NAC, dual NETosis/ferroptosis inhibition (cysteine supply may simultaneously feed ILC2 ferroptosis tolerance, both compartments must be measured)
Cholinergic nerve/α7nAChR axis [A/M; H correlation]	α7nAChR suppresses macrophage glycolysis	Inflammatory-reflex metabolic brake	Modulates stress-exacerbation pathways	Vagal modulation, breathing-training approaches

Evidence codes: H, human clinical/cohort/ex vivo tissue data; A, animal models; M, cell-based mechanistic studies. Codes are ordered by strength of available evidence. FAO/OXPHOS is not synonymous with repair. Tregs, M2-like macrophages and pathogenic ILC2s share lipid and amino-acid substrate pools but exert opposing functions. The decisive variable is substrate access under limiting conditions, not fuel preference per se.

### 3.2 Group 2 innate lymphoid cells: the dominant innate effector and its metabolic checkpoints

Group 2 innate lymphoid cells (ILC2s) are absent from most metabolism-oriented reviews of asthma, yet they are the dominant innate source of IL-5 and IL-13 in the airway and respond directly to the IL-33, TSLP and IL-25 alarmins released by airway epithelium (Section 3.4). They sit at the centre of precisely the phenomena this review addresses in children: the early-life IL-33 window, virus-triggered wheeze, and obesity-associated disease. Their metabolism is also among the best worked out of any innate lymphocyte.

ILC2 pathogenic function requires aerobic glycolysis, as shown by Monticelli and colleagues: arginase-1 (Arg1) is an ILC2-intrinsic metabolic checkpoint whose deficiency impairs polyamine biosynthesis and aerobic glycolysis, limiting ILC2 proliferation and abolishing type-2 lung inflammation (26). Lipid dependence is context-specific rather than universal: in helminth infection and nutritional restriction, ILC2s depend predominantly on fatty-acid uptake and metabolism to sustain barrier-protective IL-13 production (27), whereas in allergen-driven airway inflammation, IL-33-imprinted PPARγ- and DGAT1-driven lipid-droplet formation supports pathogenic ILC2 proliferation (28). Human data add a dichotomy: circulating naive ILC2s are OXPHOS-high and amino-acid dependent at steady state, yet switch to glycolysis and mTOR dependence for proliferation after IL-33 activation, so proliferation and effector function are metabolically uncoupled (29). Finally, PD-1 acts as a metabolic checkpoint on ILC2s, PD-1 deficiency shifts ILC2 metabolism toward glycolysis, glutaminolysis and methionine catabolism, and PD-1 agonism ameliorates airway hyperreactivity in experimental models (30).

Two features make ILC2s central to the framework of Section 6. First, they depend on the cystine-glutathione-GPX4 axis for survival under lipid-peroxidative stress, with ferroptosis tolerance licensing their pathogenic lipid metabolism (Section 2.3). Second, they are substrate competitors of the reparative populations discussed in Section 2.1: the same fatty-acid pool that feeds Treg and M2-like macrophage homeostasis can be diverted into ILC2 lipid droplets during type-2 inflammation. Whether such interventions help or harm may depend on which compartment captures the substrate, an open question we return to in Sections 5.3 and 7.

### 3.3 T cells: the effector-versus-regulatory balance as a metabolically determined variable

Th1/Th17 cells are often described as glycolytic, and Th2 cells have sometimes been assumed to rely on oxidative metabolism. This assumption is not supported by current data. Michalek and colleagues showed in 2011 that Th1, Th2 and Th17 cells are uniformly Glut1-high and glycolytic after activation, whereas Tregs prefer lipid oxidation (31). Effector-lineage differences arise instead from lipid and glutamine metabolic configuration, Th2 cells depend on fatty-acid synthase (FASN) and glutaminolysis, creating metabolic vulnerabilities for type-2 inflammation (32, 33).

mTORC2 regulates GATA3 expression. IL-4 receptor signals connect to PI3K through IRS-2. Therefore Th2 cell differentiation is partially controlled by metabolism. Pre-clinical studies show mTOR-targeting rapalogues and glutaminase inhibitors can suppress type-2 inflammation. Human paediatric data are still absent. Children’s metabolic plasticity suggests potential intervention windows, which need careful safety assessment.

The Treg-to-effector ratio, rather than absolute Treg number, is the informative variable in paediatric asthma, and both the ratio and Treg phenotypic stability are metabolically determined. Foxp3 shifts cells away from Myc-driven glycolysis toward oxidative metabolism, which is why Tregs retain function in the lactate-rich airway conditions that suppress effector T cells. This asymmetry links directly to the lactate-lactylation material of Section 2.1 and to H3, and it means that lactate is not uniformly pro-inflammatory: it is a selective pressure that reshapes the effector-versus-regulatory balance.

Under sustained metabolic stress, Treg-mediated tolerance can be abrogated under chronic Th17-biased inflammation, and Tregs themselves can acquire effector-like phenotypes, losing Foxp3 and gaining IL-17 or type-2 cytokine capacity (34, 35). This conversion supplies a concrete cellular mechanism for the Metabolic Latching Model (Section 6): a persistently glycolytic, lactate-rich airway does not merely amplify existing inflammation, it can neutralize the regulatory compartment and recruit effector-like cells into the inflammatory pool, a more direct route to a locked inflammatory state than a generic metabolism-epigenome loop.

Finally, ILC2s and Tregs interact through both suppression and substrate competition, the former experimentally demonstrated (36, 37), the latter predicted by their shared metabolic requirements (Section 3.2). These two populations share lipid and amino-acid dependence yet exert opposing functions, and their relationship in the paediatric airway is precisely where immunometabolic logic predicts this review should be most interesting, and where evidence remains thinnest.

### 3.4 Airway epithelium: more than a physical barrier for immune coordination

Airway epithelium is not only a physical barrier. It is also an important source of alarmins including IL-33, TSLP and IL-25. Epithelial metabolic status can change alarmin release. Allergen- or virus-induced epithelial glycolysis may facilitate the release of IL-33 (38, 39). Mechanical compression caused by airway smooth muscle contraction also changes epithelial metabolism through ATP release and purinergic signals (40–42). These alarmins act directly on group 2 innate lymphoid cells (ILC2s), which are the dominant innate source of IL-5 and IL-13 in the airway. Epithelial alarmin release and ILC2 activation therefore form a coupled axis that is central to the paediatric phenotypes discussed in Section 4 (Section 3.2).

Goblet-cell metaplasia and excessive mucus production consume large amounts of substrates for glycosylation. The supply of glutamine and UDP-sugar may limit mucus plug formation. This provides an angle for intervention research.

### 3.5 Eosinophils and neutrophils: two different terminal metabolic patterns

IL-5-supported mitochondrial integrity maintains eosinophil survival. Lipid metabolic states control eosinophil degranulation and release of lipid mediators. Neutrophils show strong glycolytic features and consume large amounts of NADPH during NETosis. These observations help explain the link between glutathione system damage, redox imbalance and severe neutrophilic asthma (43–45). When we distinguish eosinophilic and neutrophilic asthma endotypes, part of the difference may reflect competition between OXPHOS-lipid pathway and glycolysis-oxidative-stress pathway.

### 3.6 Airway smooth muscle: an under-studied metabolic effector

Immune cell metabolic reprogramming drives inflammation. Metabolic changes in airway smooth muscle (ASM) can directly produce airway hyperresponsiveness. This is a key clinical marker for paediatric asthma measured by bronchodilator tests.

-Remodelling-related metabolic plasticity. ASM remodelling does not fully follow the Warburg glycolysis rule. Asthmatic bronchial smooth muscle remodelling may rely heavily on fatty acid oxidation (FAO). ASM hyperplasia in severe asthma links to enhanced mitochondrial OXPHOS (46). These different metabolic patterns may correspond to early proliferation stage and late hypertrophy stage. TGF-β and PDGF signals push ASM toward synthetic phenotype through PI3K-mTOR-HIF-1α pathways (46, 47). This phenotype change can retain metabolic-epigenetic memory. Synthetic cell status can persist even after original stimuli disappear.
-Mitochondrial dysfunction in severe disease. Samples from fatal asthma show ASM cells have lower mitochondrial-membrane potential, impaired OXPHOS and higher mtROS. Mitochondrial energy disorder correlates with airway hyperresponsiveness and higher sensitivity of contractile apparatus to acetylcholine. These phenomena are similar to mitochondrial quality control changes in immune cells.
-Coupling between metabolism and contraction. Glycolysis flux affects ATP-driven calcium pump activity and myosin-light-chain phosphorylation. We propose a testable hypothesis. Basal ASM glycolysis flux may set calcium signal gain. It may contribute to AHR instead of only acting as a downstream result of inflammation.
-Drug-metabolism interaction. β2-adrenergic receptors themselves have strong metabolic regulatory functions. cAMP-PKA signals trigger glycogen decomposition, lipolysis and potassium inflow. Tolerance after long-term β2-agonist use may come from changed receptor-metabolism coupling. This supports drug-repurposing discussion in Section 5.5.

### 3.7 Mast cells: metabolic requirements for degranulation

Mast cells play key roles in allergic reaction and exercise-induced bronchoconstriction (EIB). After FcϵRI cross-linking, PI3K-mTOR-HIF-1α signals quickly increase glycolysis. Glycolysis supplies energy for cytoskeleton rearrangement, vesicle fusion and lipid mediator synthesis during degranulation. Mitochondrial ROS amplify degranulation as signal molecules, not merely toxic by-products (48, 49).

From the paediatric perspective, mast-cell effector function can be modified through metabolic targets. Glycolysis inhibition can suppress degranulation at early steps. Clinical effects of montelukast in EIB are consistent with lipid-mediator pathway modulation, but receptor antagonism downstream of mediator synthesis does not itself establish metabolic reprogramming (Section 5.2).

### 3.8 Fibroblasts: metabolic drivers of subepithelial remodelling

Subepithelial fibrosis is a major component of airway remodelling. Myofibroblasts mediate this process, but their metabolic features receive limited attention. Collagen hydroxylation and glycosylation consume large amounts of oxygen and ATP. Myofibroblasts therefore increase glycolysis to compensate. Glycolysis inhibition can reduce collagen deposition in pre-clinical asthma models.

TGF-β signal rewrites glutamine and proline metabolism. It provides direct raw materials for collagen and forms self-reinforcing signal-metabolism-matrix loops (50, 51). ASM and myofibroblast phenotypes belong to one continuous functional spectrum. Contractile-to-synthetic transformation uses overlapping PI3K-mTOR-HIF-1α signals. Interventions targeting metabolic-epigenetic memory may work better for tissue remodelling than therapies targeting only one single cell type.

## 4 Paediatric-specific biological considerations

### 4.1 Early-life developmental windows and stage-dependent biology

Fetal and neonatal alveolar macrophages are still proliferating and maturing. Their metabolic requirements differ from adult resident AMs (52, 53). Intrauterine high-glucose or high-lipid environments such as gestational diabetes and maternal obesity may change AM and Treg functional thresholds through epigenetic-metabolic mechanisms. These changes may contribute to transgenerational-like transmission of allergy and asthma susceptibility (54, 55). Caesarean delivery and perinatal antibiotic exposure also disturb microbiome-metabolism signals within this critical window. The early-life IL-33 window is of particular relevance to ILC2s. ILC2s respond directly to IL-33, TSLP and IL-25, and their metabolic programme is imprinted by these alarmins. This places ILC2s at the interface between early-life epithelial alarmin exposure and later type-2 endotype persistence. **Figure 1** summarizes these metabolic influences in developmental windows.

**Figure 1:**
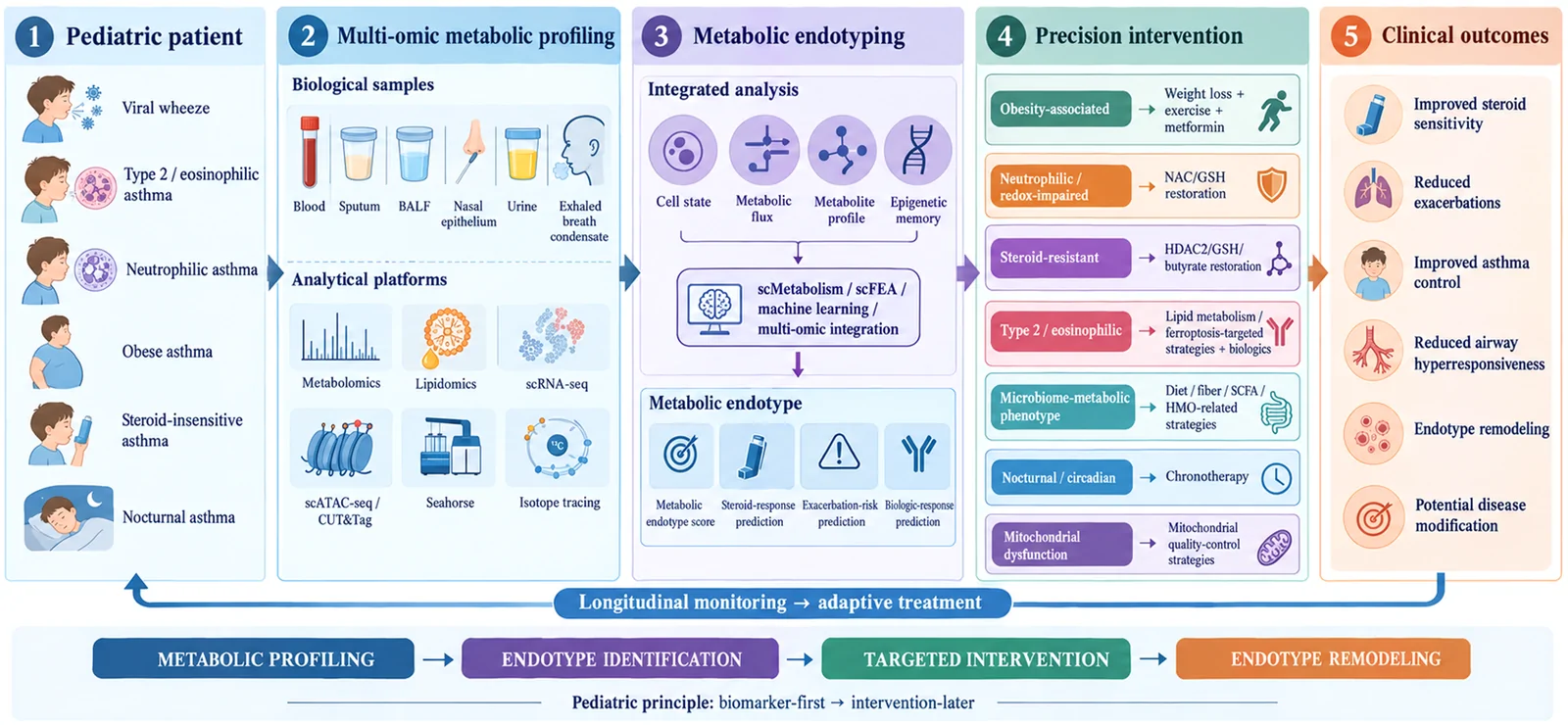
Developmental-metabolic-programming and paediatric-asthma-endotypes. Critical developmental-windows spanning prenatal-life through puberty shape immune-cell-metabolic-states in children. Environmental, infectious, nutritional, microbial, circadian and hormonal-factors modify metabolic profiles within macrophages, T-cells, eosinophils, neutrophils, airway-epithelial-cells and airway-structural-cells. These cell-specific-metabolic-programmes contribute toward partially-overlapping asthma-endotypes including type-2/eosinophilic, neutrophilic and obesity-associated phenotypes. Metabolic-phenotypes remain dynamic and potentially reversible across developmental-stages. ILC2s are shown as a key innate population linking early-life alarmin exposure to type-2 endotypes.

### 4.2 Trained immunity: possible metabolic memory in virus-wheeze-asthma progression

Respiratory syncytial virus, rhinovirus and influenza are major triggers for wheeze in young children (23). Under the trained-immunity framework, virus infection may drive metabolic reprogramming through HIF-1α/mTOR. Monocyte-macrophage cells can maintain a low-grade hyper-responsive status for weeks to months after infection. They produce stronger IL-1β/IL-6 response and Th2 bias when facing secondary virus stimulation (23, 24). RSV and rhinovirus wheeze are not only macrophage-trained-immunity phenomena. These viruses also act on airway epithelium to release IL-33, TSLP and IL-25, which directly activate ILC2s. ILC2s are therefore a second cellular route through which repeated viral wheeze may translate into persistent type-2 inflammation. Their metabolic checkpoints are discussed in Section 3.2.

This brings forward a testable clinical hypothesis. Children with repeated virus-triggered wheeze may show higher baseline glycolysis flux in peripheral blood monocytes compared with non-wheezing children. This metabolic signature may predict future asthma onset. Biomarkers reflecting metabolic-immune memory remain under-explored in paediatric cohorts.

The COVID-19 pandemic provides a natural observation opportunity. SARS-CoV-2 infection can cause obvious immunometabolic changes including enhanced glycolysis, tryptophan redirection and mitochondrial dysfunction. Clinical reports describe new wheeze or asthma-like symptoms in children after SARS-CoV-2 infection. Post-viral metabolism-epigenetic memory may contribute to post-infectious airway disease. Causal relationships still need firm proof (56–58).

### 4.3 Obese asthma: two-way interaction related to metabolic reprogramming

Among paediatric asthma subtypes, obese asthma has relatively rich evidence supporting metabolic-immune interaction (59). Adipokine changes (high leptin, low adiponectin) can reshape immune cell metabolic preference (Section 2.1). Insulin resistance and high blood glucose provide systemic metabolic background that favours glycolysis-driven inflammation. Metabolic features of adipose-tissue macrophages may mirror airway macrophage features. Whole-body immunometabolic status may partly decide local lung inflammatory phenotypes.

Obesity-associated asthma is also an ILC2-relevant endotype. Adipokines, insulin resistance and high glucose alter the lipid and amino-acid pools available to ILC2s, Tregs and M2-like macrophages. The same fatty-acid pool supports pathogenic ILC2s and reparative Tregs/M2 cells, so this endotype may depend on which population wins under conditions of limited substrate (Section 2.1).

Weight loss, diet adjustment and physical exercise may improve patient conditions by reversing metabolic reprogramming. They are not only working through improving respiratory mechanical function. The obese-asthma endotype may respond to metabolic-targeted intervention. It deserves consideration in future endotype-stratified clinical trials.

### 4.4 Microbiome-metabolism axis: tryptophan, short-chain fatty acids and secondary bile acids

Intestinal and airway microbiota influence asthma mainly through three metabolic pathways. First, short-chain fatty acids: butyrate induces colonic Treg differentiation (60), supports Treg homeostasis, adjusts FAO activity and can restore HDAC2 function linked to steroid sensitivity (Section 5.3). Second, tryptophan metabolism: balance between microbial indole products and IDO-produced kynurenine. Third, secondary bile acids: they send regulatory signals to myeloid cells through TGR5/GPR131.

Birth-cohort studies show faecal metabolite profiles measured in the first year of life correlate with asthma risk at age six (61–63). Microbiome-targeted interventions for children include probiotics, prebiotics and nutritional adjustment. To get stable clinical effects, trial design should set metabolite-related endpoints instead of only detecting microbiota composition.

### 4.5 Circadian rhythm, sleep and metabolic reprogramming: possible metabolic clock for nocturnal asthma

Nocturnal or early-morning symptom worsening is a well-known feature of paediatric asthma. Its mechanism still needs further explanation. Core circadian genes (BMAL1/CLOCK-REV-ERBα/ROR) directly control metabolic programs in immune and body cells. REV-ERBα can suppress glycolysis and inflammation-related genes. Loss of its function increases macrophage glycolysis and IL-1β production (64, 65).

Circulating cortisol rhythm couples with glucose metabolism. Early-morning cortisol reduction coincides with peak airway hyperresponsiveness. Sleep deprivation itself increases monocyte glycolysis bias and insulin resistance. Poor sleep and bad asthma control in children may link partly through these immunometabolic pathways (66).

Chronotherapy adjusts medicine administration time according to circadian phase. The different clinical performance of slow-release theophylline taken every night supports this idea. Chronotherapy can be regarded as a kind of metabolic-window-oriented intervention. Metabolic reprogramming theory provides a hypothetical mechanism for time-adjusted medication in paediatric asthma. This idea needs clinical verification. Few existing reviews systematically discuss this topic.

### 4.6 Puberty, sex hormones and asthma sex-related changes

Epidemiology shows asthma prevalence is higher in boys before school age. This pattern reverses after puberty (67, 68). Sex hormones directly affect immunometabolism. Oestrogen can improve mitochondrial function and fatty acid oxidation through ERα signals. It may increase mast-cell degranulation tendency and Th2-biased immune response. Testosterone generally suppresses immune response. Insulin sensitivity changes in puberty overlap with rising obesity rates. They create a second developmental window for metabolic-immune programming.

These pathways may partly explain why some childhood wheeze disappears at puberty, while some girls get new or worse asthma in adolescence. Pre-puberty and post-puberty periods are additional important windows for metabolic-targeted research. Sex-stratified analysis should be encouraged in paediatric asthma-metabolism studies.

### 4.7 Neuro-metabolic-immune axis: cholinergic signal and psychological stress

Metabolic reprogramming receives influence not only from environmental stimuli but also from nerve output. Existing reviews about asthma-metabolism rarely cover this topic. It has relevance for paediatric research because caregiver stress, children anxiety and asthma control connect closely.

The cholinergic anti-inflammatory reflex works in the following way. Vagus nerve releases acetylcholine. Acetylcholine acts on macrophage α7nAChR receptors. It suppresses glycolysis as well as NF-κB/NLRP3-driven IL-1β and IL-6 production (69, 70). Parasympathetic nerve function serves as a homeostatic tuner for immune cell metabolism. Asthmatic children show autonomic imbalance. Their vagal activity reduces while airway cholinergic hyper-reactivity appears. This may mean loss of metabolic braking function.

Psychological stress activates HPA axis and sympathetic-catecholamine response. It drives glycolysis bias and mobilizes lymphoid-myeloid immune cells. Stress-related asthma exacerbations in children may partly come from this neuro-metabolic-immune cascade (71, 72). Autonomic nerve activity itself follows circadian rhythm. It provides nerve-related explanation for nocturnal asthma mentioned in Section 4.5. Non-invasive vagal modulation and mind-body breathing training can be used as low-risk test tools for metabolic-reprogramming research in children.

### 4.8 Metabolomic endotyping: from Th2 markers to metabolic fingerprints

Paediatric-asthma metabolomics studies show blood, urine and exhaled-breath-condensate metabolite profiles can distinguish asthmatic versus control participants. These profiles may also predict exacerbation risk and treatment responsiveness. Relevant analytes include histidine, polyunsaturated fatty-acid species and TCA-cycle intermediates (73).

Future work may increasingly integrate multi-omics endotyping. It combines transcriptomic cell-state read-outs, metabolomic functional measurements and epigenomic memory traces. Such composite signatures could improve predictions for natural-history trajectories and therapeutic response compared with isolated Th2-related biomarkers. **Figure 2** visualises metabolomic-based endotyping and its potential application for intervention.

**Figure 2:**
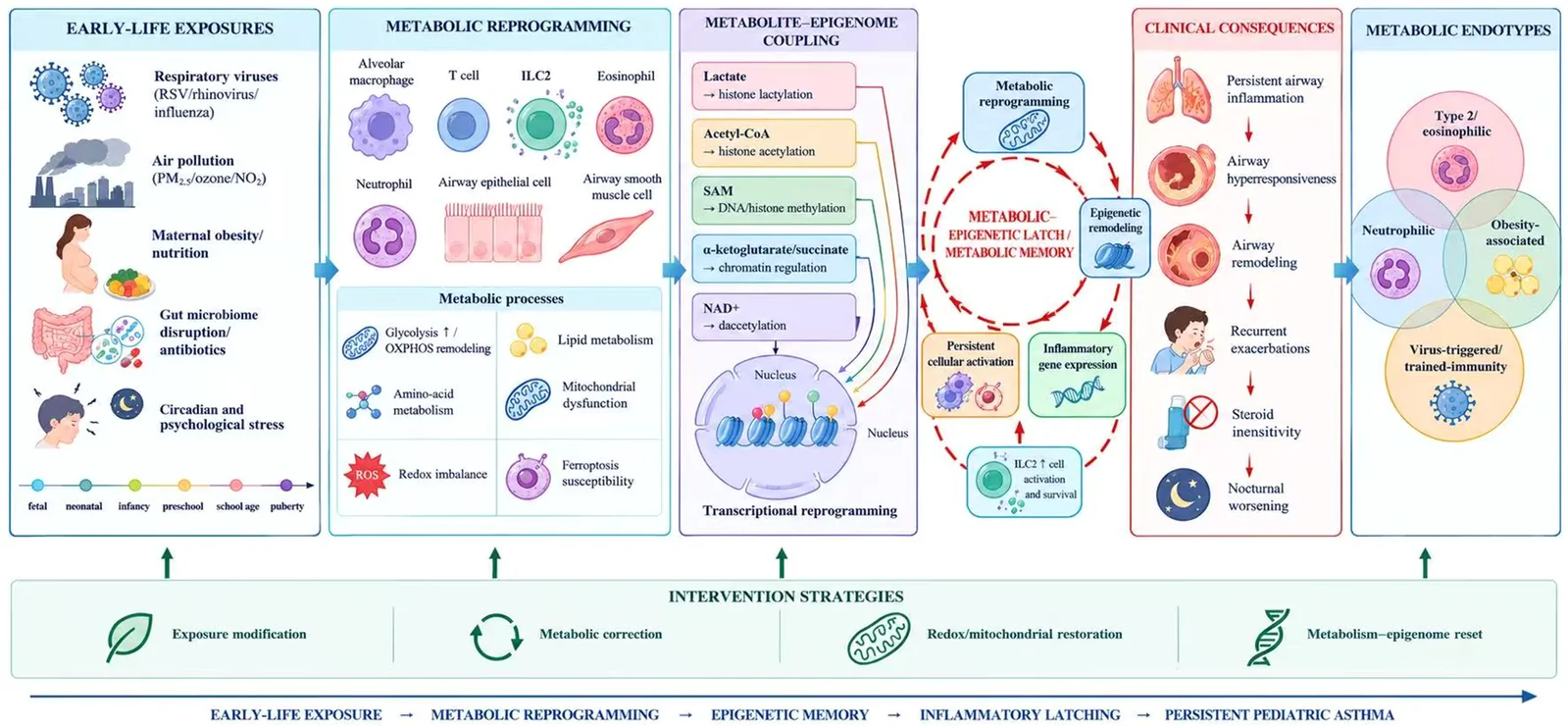
Metabolomic-based-endotyping and precision-intervention-concepts for paediatric-asthma. Integration of clinical-phenotypes with metabolomics, lipidomics, transcriptomics, epigenomics and single-cell-metabolic-profiling may enable identification of metabolically-defined-asthma-endotypes. Such multi-omic-profiles could help predict exacerbation-risk, glucocorticoid-responsiveness and general-treatment-outcomes. Potential-targeted-intervention-strategies include weight-management-programmes, redox-homeostasis-restoration, microbiome-directed-therapies, mitochondrial-supporting-agents and chronotherapy-approaches. Longitudinal-metabolomic-profiling may facilitate adaptive-treatment-adjustment and evaluation of endotype-remodelling-effects.

## 5 Metabolic targets and therapeutic strategies

### 5.1 The landscape of drug targets

**Table 2** summarises each target together with its assumed site of action, because applicability in children cannot be assessed without stating where the intervention is expected to act.

**Table 2:** Landscape of metabolic-intervention targets and preliminary paediatric-applicability assessment.

Target/pathway	Representative strategy	Assumed site of action	Paediatric applicability assessment
HIF-1α/glycolysis	2-DG, LDHA inhibition	Airway immune and epithelial cells; systemic delivery, local partitioning unmeasured	Preclinical stage; broad glycolysis suppression carries infection risk, particularly concerning in children
mTOR	Rapalogues, metformin (AMPK activation)	Systemic immune cells with indirect airway exposure; airway partitioning unmeasured	Metformin has an ample safety record and is the most realistic candidate for paediatric metabolic intervention (compartment-specific; effects on ILC2/M2 substrate competition must be evaluated)
Leukotriene pathway	Montelukast (clinically approved)	Airway (CysLT1 on airway smooth muscle and immune cells); oral dosing with documented lung exposure	CysLT1 receptor antagonist downstream of mediator synthesis. Efficacy in EIB does not establish a metabolic reprogramming property. Small-sample paediatric evidence exists.
Redox/NADPH (including ferroptosis-defence machinery)	NAC, glutathione precursors, NAD+ precursors, GPX4 induction	Dual compartment: systemic cysteine-GSH supply plus airway epithelium and neutrophils	Small-sample paediatric evidence exists; targeting glutathione cycle collapse in the neutrophilic endotype may be of particular interest. Caution: cysteine-glutathione supply also sustains ferroptosis tolerance in pathogenic ILC2s; compartment-specific monitoring is required.
Mitochondrial quality control	Mitophagy-inducing agents (urolithin A, spermidine), PINK-pathway modulators	Undetermined, no clinical delivery route established	Mechanistically blank territory; limited human safety data; high-risk high-reward direction (compartment-specific; effects on ILC2/M2 substrate competition must be evaluated)
Inflammasome	NLRP3-inhibitor compounds (dapansutrile, MCC950-class)	Systemic myeloid cells (oral bioavailability demonstrated in Phase 2); airway partitioning unmeasured	Adult Phase 2 trials conducted or ongoing (dapansutrile, open-label proof-of-concept in gout; programmes in osteoarthritis, heart failure, COVID-19 and type 2 diabetes); no paediatric data (74)
Arginine/NO axis	ARG1 inhibition, inhaled-NO modulation	Airway (inhaled NO) or systemic myeloid compartment (ARG1 inhibition)	Attractive mechanism, insufficient clinical validation
Tryptophan-IDO axis	Dietary tryptophan manipulation, IDO-modulating agents	Gut-liver axis and systemic immune compartment	Logically coherent in combination with microbiota interventions
Metabolic-epigenetic coupling	Lactylation- or HDAC-modulating compounds	Undetermined, preclinical concept	An appealing long-term strategy: directly erasing metabolic memory

Site-of-action entries state the compartment in which each target is assumed to operate; “airway partitioning unmeasured” indicates that local drug concentration at the disease site has not been quantified in humans, the compartment mismatch constraint discussed in Section 7 item 2.

### 5.2 Nutrition and lifestyle: the first battlefield of paediatric metabolic intervention

Compared with new drugs, the most realistic metabolic interventions for children come from nutrition and lifestyle. Dietary and exercise habits are malleable, and ethical barriers are low.

Vitamin D modulates AMPK–mTOR and immunometabolism. Cohort studies link gestational or early-life vitamin D status to asthma risk, but supplementation trial results are inconsistent. VDAART missed its primary endpoint for asthma or recurrent wheeze at three years (75). Reference 76 is a *post-hoc* combined analysis of two trials, not a confirmatory primary analysis. The inconsistency may arise from failure to stratify by metabolic endotype.

Omega-3 polyunsaturated fatty acids, promoting inflammation resolution through the resolvin/protectin pathway, with maternal fish-oil intervention showing reduced offspring wheeze risk in some trials (76, 77).

The dietary fibre-butyrate axis, butyrate inducing Tregs and serving as an HDAC2 substrate directly linking to steroid sensitivity.

Human milk oligosaccharides (HMOs), shaping infant microbial metabolic output (acetate, lactate, indolelactate) as the most natural carrier of metabolic programming intervention (78–80).

Exercise is a double-edged sword. Regular aerobic training improving systemic immunometabolism (reducing monocyte glycolytic bias), while acute vigorous exercise triggers bronchoconstriction. EIB is itself a metabolism-osmolarity-mediator event. The efficacy of leukotriene antagonists in EIB reflects lipid-mediator pathway modulation rather than proof of metabolic reprogramming (81, 82).

Practical recommendation: future paediatric dietary intervention trials should use immune-cell metabolomics rather than symptom scores alone as intermediate endpoints, with endotype-stratified randomization, especially for obese and neutrophilic subgroups.

### 5.3 The metabolic roots of glucocorticoid resistance: paediatric mechanisms first

Steroid resistance in childhood asthma is not a single entity, and the mechanisms should be ordered by the paediatric evidence rather than imported from adult disease. Severe therapy-resistant asthma in children is predominantly eosinophilic and type-2 in character, and its best-supported resistance mechanism operates directly on ILC2s: the TSLP-IL-7Rα-STAT5 axis. TSLP renders lung ILC2s corticosteroid resistant through STAT5 and Bcl-xL, reversible with anti-TSLP or pimozide (83). In humans, BAL ILC2s from patients with severe asthma are steroid resistant, BAL TSLP correlates with that resistance, and MEK, JAK and STAT5 inhibition reverse it (84). Notably, dexamethasone itself upregulates IL-7Rα and amplifies the TSLP signal, a treatment-induced latch that is precisely the kind of self-reinforcing mechanism this review argues should be sought in paediatric asthma (Section 6).

A second, mechanistically distinct layer of steroid resistance is well established in adult COPD and smoking-related disease and is most relevant to neutrophilic or obese paediatric subgroups. Its molecular core is impaired GR transcriptional complex function: oxidative stress activates PI3Kδ and causes oxidative inactivation and degradation of HDAC2, histone acetylation becomes unchecked, and GR-mediated anti-inflammatory transcription shuts down, the mechanism by which NAC restores steroid sensitivity in severe adult asthma/COPD (85, 86). HDAC2 activity depends on acetyl-CoA and GSH supply, and butyrate supplementation or elevated epithelial GSH restores steroid response in animal models (86). Macrolides exert part of their anti-inflammatory effect through mTOR inhibition and macrophage metabolic modulation rather than antibiosis, an explanation for their efficacy evidence in neutrophilic asthma (87, 88). Under insulin resistance, GR signalling cross-talks with enhanced PI3K pathways, a candidate mechanism for poor steroid response in obese asthma (59). However, the same cysteine-glutathione supply that restores redox defence may feed pathogenic ILC2s, whose ferroptosis tolerance depends on the cystine-GSH-GPX4 axis (12). Systemic cysteine delivery could therefore rescue one compartment while feeding the other, a tension that must be measured, not assumed away. The clinical translation proposition: steroid-sensitization regimens that restore metabolism-epigenome homeostasis (NAC, macrolides, butyrate, mitochondrial support) should be re-trialled in neutrophilic/obese childhood asthma, old drug combinations with a new mechanism, feasible in paediatrics, provided ILC2 compartments are monitored alongside.

### 5.4 Metabolic predictors in the biologics era

Anti-IL-5 and anti-IL-5Rα target a cytokine produced constitutively by airway ILC2s (Section 3.2). Anti-IL-4Rα blocks IL-4/IL-13 signalling on ILC2s as well as on T and B cells. Tezepelumab (anti-TSLP), approved for severe asthma from age 12 years, blocks TSLP upstream of ILC2 activation and is the closest clinical approximation of the mechanism proposed here (89). Anti-IL-5/IL-4Rα/IL-5Rα biologics have transformed severe asthma treatment, yet a considerable proportion of children respond poorly. Metabolomic features (baseline tryptophan metabolites, lipid mediator profiles, ferroptosis markers such as 4-HNE/MDA) may predict treatment response and monitor endotype conversion. Whether biologics, beyond suppressing inflammation, simultaneously reverse metabolic reprogramming is a novel follow-up research question (90, 91).

### 5.5 The metabolic footprint of drugs: a unified perspective on repurposing

A practical dividend of the metabolic reprogramming framework is a unified mechanistic reinterpretation of existing paediatric asthma drugs. Most already work partly through metabolic reprogramming.

Inhaled corticosteroids (ICS), beyond genomic effects, suppress immune cell glycolysis by downregulating mTOR/HIF-1α, promote oxidative metabolism and Treg-like phenotypes, and remodel lipid metabolism through mTOR-related nodes (92). Their systemic side effects, most notably growth deceleration, which persists as reduced adult height, remind us that the metabolic footprint is double-edged (93).

Long-acting β2-agonists (LABA), beyond smooth muscle relaxation, deliver a metabolic instruction through cAMP–PKA signalling to lipolysis and glycogenolysis. ICS–LABA synergy partly reflects dual coverage of contraction–metabolism targets (Section 3.6).

Low-dose theophylline, at sub-bronchodilator doses, selectively inhibits PI3Kδ and restores oxidatively-inactivated HDAC2, reversing steroid resistance (94). However, the TWICS trial in COPD (1,578 participants) found that low-dose theophylline added to inhaled corticosteroids did not reduce exacerbations (95). This negative result limits the claim that theophylline is the most representative clinical example of a metabolism-epigenome drug.

Leukotriene receptor antagonists, lipid mediator metabolic intervention (Section 5.1). Their efficacy in EIB does not establish a metabolic reprogramming property; blocking CysLT1 is receptor antagonism downstream of mediator synthesis.

Mast cell membrane stabilizers (ketotifen, cromolyn), whose effects on FcϵRI-glycolysis coupling deserve re-examination (Section 3.7).

The proposition of this section: rather than waiting for new metabolic drugs, systematically pursue metabolic mechanism repositioning of existing drugs. Clear mechanisms, available paediatric populations, and low ethical barriers make this the fastest route for the metabolic reprogramming framework to deliver clinical value.

## 6 Working model and testable hypotheses: core conceptual proposal of this review

### 6.1 The metabolic latching model (testable working model):

We propose a hypothetical “Metabolic Latching Model” as a conceptual framework requiring further experimental validation. Early-life environmental-exposures (viral infections, air pollutants, obesity, microbiome dysbiosis, circadian disruption) may induce metabolic reprogramming: shifts towards glycolytic bias, lipid-pathway re-routing, elevated ferroptosis susceptibility and compromised mitochondrial-quality-control function. Metabolic intermediates drive epigenetic remodelling events including lactylation, acetylation and methylation. A self-reinforcing metabolism-epigenetic positive-feedback loop could then lock inflammatory endotypes into persistent states even after disappearance of the original triggering stimulus. Clinically, this may manifest as persistent asthma, glucocorticoid insensitivity and nocturnal-exacerbation-prone disease trajectories. A schematic overview is presented in **Figure 3**. Operationally, we define a latched state by three criteria: (i) the original exposure has been removed; (ii) the metabolic and epigenetic state persists beyond the turnover time of the responding cells, under a pre-specified time threshold; and (iii) the state cannot be explained by continuing exposure. Cross-sectional or short-interval measurements cannot distinguish a latched state from ongoing exposure or slowly resolving inflammation, all six hypotheses below must therefore incorporate an exposure-removal interval, and this requirement is reflected in **Table 3**.

**Figure 3:**
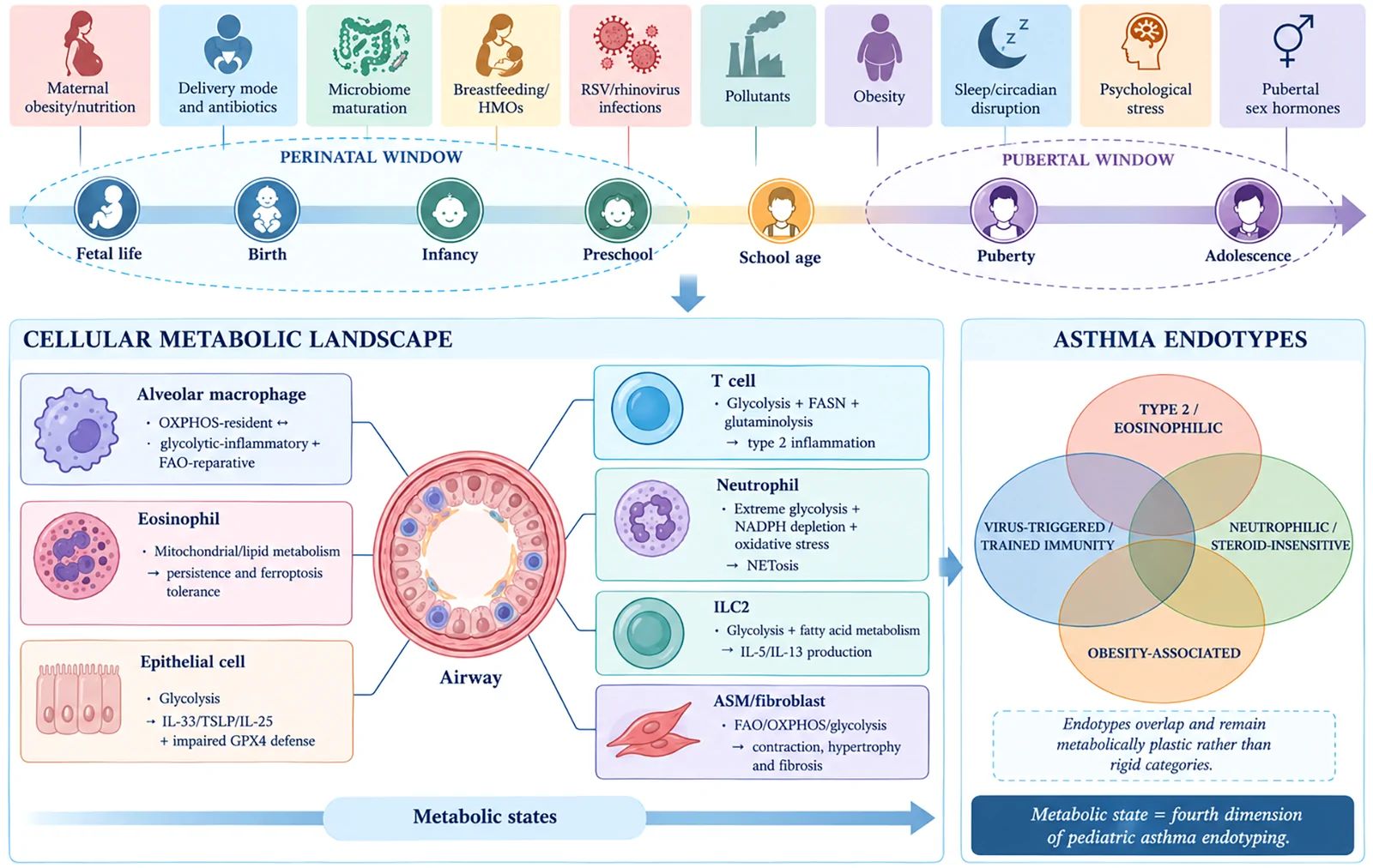
The metabolic latching model of paediatric asthma. Early-life-exposures including viral-infections, air-pollutants, maternal-metabolic-factors, microbiome-disruption, obesity and circadian-disturbance induce metabolic-reprogramming in immune and airway-structural-cells. Shifts in glycolysis-oxidative-phosphorylation balance, lipid-metabolism, mitochondrial-function and redox-homeostasis drive metabolic-epigenetic-coupling via intermediates such as lactate, acetyl-CoA and NAD +. These interactions establish self-reinforcing metabolism-epigenetic-feedback-loops that may sustain inflammatory states even after initial-trigger-removal. This may contribute to asthma chronicity, airway-hyperresponsiveness, tissue-remodelling and glucocorticoid-insensitivity. ILC2s are included as a central innate source of IL-5/IL-13 and as a population whose lipid and amino-acid metabolism overlaps with Tregs and M2-like macrophages, illustrating the substrate competition discussed in Section 2.1.

**Table 3:** Verification design overview for the six testable hypotheses of the metabolic latching model.

Hypothesis	Core prediction	Verification methods	Required samples	Feasibility rating
H1: monocyte glycolytic baseline predicts wheeze-to-asthma transition	Baseline glycolytic flux of peripheral monocytes is elevated in children with recurrent viral wheeze versus non-wheezing controls and predicts conversion	Seahorse XF cell energy metabolism analysis; single-cell metabolic pathway activity scoring (scMetabolism); longitudinal sampling around infections and during a defined virus-free interval	200–300 cases in a prospective birth/wheeze cohort, 3–5 years follow-up	★★★★ (minimally invasive, single blood draw, controllable cost)
H2: AM glycolytic-inflammatory subsets correlate negatively with persistence and steroid response	High proportions of glycolytic-inflammatory AMs indicate persistent asthma and poor steroid response; emergence of reparative AMs predicts remission	BALF scRNA-seq plus scMetabolism pathway activity scoring; Seahorse validation of sorted AMs; flow cytometric phenotyping; exposure-removal follow-up interval required	50–100 BALF samples (retrospective plus prospective)	★★★ (bronchoscopy required, moderate ethical threshold; public single-cell data allow preliminary verification)
H3: the lactate-lactylation axis is necessary for neutrophilic endotype self-maintenance	Inhibiting lactylation or clearing lactate disrupts the self-maintenance of the neutrophilic endotype, synergizing with NAC	Ex vivo sputum neutrophils: NETosis inhibitors plus/minus NAC; histone lactylation Western/CUT&Tag; lactate production/output blockade; include ILC2 viability/ferroptosis-tolerance and ILC2-to-Treg ratio readouts alongside neutrophil endpoints; exposure-removal interval required to distinguish latching from persistent inflammatory exposure	30–50 induced sputum samples of neutrophilic asthma	★★★ (sputum readily available, mechanistic readouts clear)
H4: metabolic intervention in obese asthma can reverse the endotype	After weight loss plus metformin, peripheral and airway immune cell metabolic programs swing back toward OXPHOS/reparative states rather than merely improving pulmonary mechanics	Randomized controlled trial; pre/post Seahorse plus targeted metabolomics (blood/induced sputum) with lung function and symptom scores; post-intervention follow-up must include an exposure-free interval	60–100 obese asthmatic children, 16–24 weeks intervention	★★★★ (old drug plus lifestyle intervention, ethics-friendly, advantage of the childhood plasticity window)
H5: circadian amplitude determines nocturnal symptoms and chronotherapy efficacy	Low clock-gene amplitude or phase shift correlates with severe nocturnal symptoms; phase-timed dosing outperforms fixed-time dosing	Peripheral blood qPCR of clock-gene rhythms sampled every 4 h; wrist actigraphy; chronopharmacology-stratified subgroup; circadian phase sampling repeated after exposure-removal follow-up interval	40–60 cases, 48 h intensive sampling	★★ (heavy sampling burden, paediatric compliance challenge; adult verification first is acceptable)
H6: the HDAC2-GSH/butyrate axis determines steroid sensitivity	Epithelial HDAC2 activity correlates positively with GSH/butyrate levels in steroid-insensitive children; butyrate precursor supplementation partially restores GR function	Airway epithelial organoids plus/minus butyrate and dexamethasone; HDAC2 activity fluorometric assay; GR target-gene transcription readouts; steroid washout follow-up required to test whether HDAC2/GR function reverts after dexamethasone removal	30–50 cases (nasal mucosa/induced sputum-derived epithelium), organoid culture	★★★★ (pure *in-vitro*/ex vivo path, no systemic dosing risk, fast translation)

Not all six designs need to begin at once. We propose a first wave of three, selected by the criteria of feasibility, minimal participant burden, and leverage of current technology. H1 requires only a single blood draw plus structured viral-event histories, and its peripheral monocyte readout doubles as a test of the surrogate-compartment question raised in Section 7. H4 re-uses an approved drug (metformin) and a lifestyle intervention in a population where both are independently indicated, and directly tests the model’s central prediction, that endotype remodelling is achievable in children. H6 is entirely ex vivo, carries no systemic dosing risk, and tests the treatment-induced latch mechanism of Section 5.3 with organoid technology already standard in paediatric respiratory laboratories. Together these three span the accessible-to-site sampling hierarchy, blood, whole-body intervention, airway-derived tissue, so their results cross-validate one another before the bronchoscopy-dependent designs (H2, H3) are attempted. The number of asterisks indicates the level of importance of each item, with more asterisks representing higher importance.

### 6.2 Six empirically testable hypotheses are derived from this conceptual framework

-H1: Baseline glycolytic flux of peripheral blood monocytes (Seahorse or single-cell metabolic pathway activity scoring) predicts the transition from recurrent wheeze to asthma; a clean post-infection interval with no further viral events must be specified, because only measurements after exposure removal can separate a latched state from continuous re-exposure;
-H2: The proportion of glycolytic inflammatory-state AM subsets correlates negatively with asthma persistence and steroid response (testable on BALF single-cell data);
-H3: The lactate-lactylation axis may participate in the self-maintenance of the neutrophilic endotype, but NAC/glutathione precursors may simultaneously support pathogenic ILC2 survival via the cystine-GSH-GPX4 axis. Ex vivo sputum neutrophils and BAL ILC2s should be studied in parallel; NETosis inhibition plus/minus NAC should include ILC2 survival, IL-5/IL-13 output and the ILC2-to-Treg ratio as safety endpoints (Section 2.1 caveat; Section 3.3).
-H4: Metabolic intervention for obese asthma (weight loss plus metformin) can remodel the endotype by reversing the metabolic programs of peripheral and airway immune cells, rather than merely improving pulmonary mechanics;
-H5: The expression phase of core circadian clock genes (e.g., peripheral blood REV-ERBα amplitude) correlates with nocturnal symptom severity in childhood asthma and may influence the efficacy of nighttime intervention;
-H6: Airway epithelial HDAC2 activity in steroid-insensitive children correlates positively with GSH/butyrate metabolic levels, and butyrate precursor supplementation can partially restore steroid sensitivity (testable with organoids plus ex vivo GSH measurement).

Single-cell verification paths for this framework: the above hypotheses are now technically testable. Metabolic pathway activity scoring of BALF/sputum scRNA-seq data with scMetabolism (96), supplemented by flux inference from scFEA (97), locates every cell simultaneously in a two-dimensional cell-type-by-metabolic-state space. These tools score metabolic pathway gene expression, not flux. Isotope tracing is a flux measurement and is listed separately in Section 7. Adding scATAC or CUT&Tag directly observes metabolism–epigenome coupling and pins down the key regulatory nodes of the latching loop. The predicted transcriptional overlap between AMs and T cells under inflammatory conditions in BALF must not be interpreted as a functional continuum until doublet detection, ambient correction and protein-level confirmation are performed. Moving this framework from hypothesis to atlas will require the accumulation, integration, and sharing of paediatric asthma single-cell-metabolism-epigenome multi-omics data.

## 7 Translational bottlenecks and research agenda

### 7.1 Correlation versus causation

Most evidence is associational. Lineage-specific conditional knockout models of metabolic enzymes and human organoid validation are still scarce; supporters note that metabolic interventions (2-DG, metformin) alter disease outcomes, but with insufficient specificity.

### 7.2 Spatiotemporal resolution and access to the site of action

Metabolites have short half-lives and steep local concentration gradients; technological gaps are now being filled rapidly (see the toolbox below). A related and arguably harder constraint is compartment mismatch. The measurements most easily obtained in children, blood cells and plasma metabolites, sample a surrogate compartment, whereas the disease acts in the airway wall. Site-of-action tissue (BALF cells, bronchial biopsies and brushings) requires procedures restricted in children, particularly longitudinally. Nasal epithelium and induced sputum are practical compromises whose validity as bronchial surrogates is only partially established (98). An explicit prerequisite for the field is validation of which peripheral metabolic signatures track airway cell states; this requirement is built into **Table 3**, where H1 pairs a peripheral monocyte readout with clinical conversion, an explicit test of whether peripheral signatures track disease, and H2 is designed to sample the airway compartment directly, with the ethical and practical constraints stated in the table.

### 7.3 Scarcity of paediatric evidence and dynamism of the phenotype

Most immunometabolism studies use adult cells and mice; prospective metabolic cohorts of childhood BALF, induced sputum, and blood remain scarce. The paediatric study population adds a second constraint: the phenotype itself moves. Lung function, immune repertoires and metabolic set-points change with age; wheeze phenotypes remit and relapse around infections; and growth confounds any longitudinal readout not indexed to age and pubertal stage. Designs must treat phenotype conversion as an expected event rather than noise, with repeated characterisation and pre-specified re-classification rules, and power calculations should assume conversion and attrition rates far above those of adult trials.

### 7.4 Ethics of intervention windows

Modulating immune metabolism in children carries developmental safety concerns, requiring a biomarker-first, intervention-later stepwise path.

### 7.5 Standardization of nomenclature and endotyping

Future studies should report immune cell metabolic scores (scMetabolism/ssGSEA), key metabolite fluxes, and epigenetic markers simultaneously to enable cross-study integration.

### 7.6 Methodological toolbox

Single-cell layer: scRNA-seq plus metabolic pathway activity scoring with scMetabolism and flux inference from scFEA, combined with scATAC to lock metabolism–epigenome cell states, and spatial transcriptomics to localize metabolic subsets anatomically.

Metabolite layer: targeted/untargeted LC-MS (blood, urine, EBC, BALF), lipidomics, and isotope tracing (13C-glucose/glutamine) as a direct flux measurement, distinct from the computational scores and inference above.

Functional layer: Seahorse cell energy metabolism analysis, NAD(P)H and mitochondrial membrane potential live imaging, ferroptosis probes (C11-BODIPY).

Model layer: air-liquid interface epithelial organoids, macrophage-epithelium-microbiota co-culture, organoid-on-chip exposure to viruses/pollutants; Computational layer: machine-learning multi-omic endotyping and digital-twin-style metabolic flux modeling (COBRA/flux balance).

Clinical layer: nested metabolomic sub-studies in birth cohorts, metabolic dynamics sampling around viral challenge tests, and chronopharmacology designs with circadian-phase sampling.

### 7.7 Controversies and open questions

Causation. Most evidence is associational. There are few causal validations in asthma phenotypes of lineage-specific metabolic enzyme knockouts. Metabolic interventions alter outcomes, but non-specifically.

*In-vivo* applicability of the Warburg dichotomy. Most M1/M2 and Th1/Th2 metabolic dichotomies derive from *in-vitro* polarization conditions. *In-vivo* airway metabolic programs form a continuum and are highly reversible.

Latching reversibility. Whether latching is truly irreversible, and under what conditions it can be reversed, lacks long-term tracking data.

Trained immunity evidence. Human evidence relies heavily on ex vivo restimulation experiments. Its *in vivo* persistence and clinical significance are still debated. Longitudinal validation in paediatric cohorts, infection to metabolic markers to asthma conversion, remains scarce.

Stability of metabolomic endotypes. Metabolites are strongly influenced by diet, circadian rhythm, and medications, and the diurnal and within-day variation of metabolic fingerprints may exceed between-endotype differences. This supports the necessity of time-standardized sampling (Section 4.5).

Specificity paradox of intervention. The more upstream the metabolic target (mTOR, HIF-1α), the more universal but also the more likely to interfere with the normal metabolic plasticity required for growth and development in children, a paediatric-specific safety constraint that cannot be extrapolated from adult studies. Interventions that push cells toward oxidative metabolism, AMPK activation, mitophagy induction, dietary strategies, must be evaluated for compartment-specific effects. The same fatty-acid pool supports Tregs, M2-like macrophages and pathogenic ILC2s; whether such interventions help or harm may depend on which population captures the substrate in an inflamed airway.

## 8 Conclusion

Studies of paediatric asthma are exploring perspectives beyond describing over-expressed-cytokines, toward investigating persistently-altered cellular-metabolic-programmes. A metabolic-reprogramming-oriented conceptual framework can integrate environmental-exposure-effects, microbiome-related influences, nutrition, circadian-biology and genetic susceptibility. It provides one plausible mechanistic explanation for how early-life-environmental-insults can shape later-childhood-disease-manifestations. Mechanistic themes including ferroptosis-related-pathways, mitochondrial-homeostasis, metabolic-origins of glucocorticoid-resistance and circadian-immunometabolic-coupling help connect molecular-level observations to paediatric clinical contexts. Children retain greater immune-metabolic-plasticity relative to adults. Metabolism-oriented-interventions may support endotype-remodelling, which may be harder to achieve in older-patient-populations. To move from synopsis to research programme, we nominate the first-wave studies described in Section 6 as feasible starting points that test the model’s central predictions with current technology. The “Metabolic Latching Model” together with its six derived-testable-hypotheses await further empirical assessment driven by advancing single-cell-multi-omics-technologies.
